# iM-Seeker: a webserver for DNA i-motifs prediction and scoring via automated machine learning

**DOI:** 10.1093/nar/gkae315

**Published:** 2024-04-27

**Authors:** Haopeng Yu, Fan Li, Bibo Yang, Yiman Qi, Dilek Guneri, Wenqian Chen, Zoë A E Waller, Ke Li, Yiliang Ding

**Affiliations:** Department of Cell and Developmental Biology, John Innes Centre, Norwich Research Park, Norwich NR4 7UH, UK; Department of Computer Science, University of Exeter, Exeter EX4 4QF, UK; Department of Cell and Developmental Biology, John Innes Centre, Norwich Research Park, Norwich NR4 7UH, UK; Department of Cell and Developmental Biology, John Innes Centre, Norwich Research Park, Norwich NR4 7UH, UK; School of Pharmacy, University College London, 29-39 Brunswick Square, London WC1N 1AX, UK; School of Pharmacy, University College London, 29-39 Brunswick Square, London WC1N 1AX, UK; School of Pharmacy, University College London, 29-39 Brunswick Square, London WC1N 1AX, UK; Department of Computer Science, University of Exeter, Exeter EX4 4QF, UK; Department of Cell and Developmental Biology, John Innes Centre, Norwich Research Park, Norwich NR4 7UH, UK

## Abstract

DNA, beyond its canonical B-form double helix, adopts various alternative conformations, among which the i-motif, emerging in cytosine-rich sequences under acidic conditions, holds significant biological implications in transcription modulation and telomere biology. Despite recognizing the crucial role of i-motifs, predictive software for i-motif forming sequences has been limited. Addressing this gap, we introduce ‘iM-Seeker’, an innovative computational platform designed for the prediction and evaluation of i-motifs. iM-Seeker exhibits the capability to identify potential i-motifs within DNA segments or entire genomes, calculating stability scores for each predicted i-motif based on parameters such as the cytosine tracts number, loop lengths, and sequence composition. Furthermore, the webserver leverages automated machine learning (AutoML) to effortlessly fine-tune the optimal i-motif scoring model, incorporating user-supplied experimental data and customised features. As an advanced, versatile approach, ‘iM-Seeker’ promises to advance genomic research, highlighting the potential of i-motifs in cell biology and therapeutic applications. The webserver is freely available at https://im-seeker.org.

## Graphical Abstract

**Graphical Abstract:**
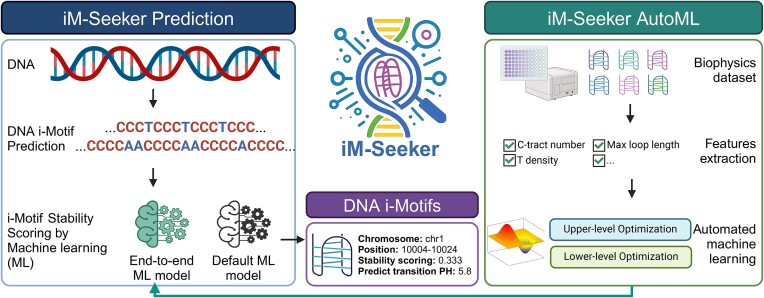

## Introduction

Beyond the canonical B-form double helix, DNA can assume various alternative conformations, including the triplexes, cruciforms, G-quadruplexes, and i-motifs. These conformations, collectively referred to as non-B DNA structures, can form during cellular processes like replication and transcription (1). In 1993, researchers identified that DNA sequences abundant in cytosines could transition into the i-motif, a form distinct from the standard B-DNA, when exposed to acidic environments (2,3). This transformation is facilitated by a distinct hemiprotonation process of cytosine-cytosine pairings, culminating in the intertwined C·CH(+) pattern and a signature quadruple-helical structure. The emergence of this structure is predominantly influenced by specific types of cytosine-rich DNA sequences, typically marked by clusters of cytosines interspersed with a limited number of other nucleotides (3).

The i-motif has increasingly drawn attention due to its presumed involvement in a range of complex biological functions. It has been suggested that the i-motif functions as a molecular switch in gene expression, a fundamental biological process, and that specific small molecules can target the dynamic balance between the i-motif and the flexible hairpin to influence this expression (4). This highlights the potential therapeutic applications of targeting the i-motif for controlling gene expression (5). Beyond gene expression, connections have been drawn between the i-motif and telomeric DNA, which is essential for maintaining chromosomal stability. Furthermore, its stability under mildly acidic conditions insinuates its possible involvement in cellular functions within such environments (6). Overall, the i-motif's multifaceted roles encompass gene expression modulation, telomere biology, and chromosomal maintenance, emphasizing its significance in cell biology and its potential as a therapeutic target (7).

i-Motif structures, have been probed using a plethora of techniques (8). While nuclear magnetic resonance (NMR) spectroscopy and X-ray diffraction offer insights into their unique proton spectra and high-resolution structures. UV molecular absorption spectroscopy stands out for its routine diagnostic potential. This method observes hyperchromicity between 275 to 300 nm during cytosine protonation and monitors absorbance shifts in the 275–295 nm range upon temperature or pH variations (9,10). Importantly, the transition pH, where half the population exists as i-motif and half as random coil, is especially informative about the i-motif's stability and formation conditions (8). Other corroborative methods include circular dichroism (CD), which shows characteristic i-motif bands, synchrotron radiation circular dichroism (SRCD) for a nuanced look at base protonation, fluorescence techniques, such as FRET, and mechanical techniques like Laser Tweezers (11–14). Additional methods, such as PAGE and SEC for structural differentiation, Raman spectroscopy for protonation detection, and mass spectrometry have expanded our understanding of these captivating structures (15).

Currently, there is a scarcity of predictive software specifically designed for i-motifs, particularly in terms of effective scoring systems. While tools such as G4-iM Grinder have the capability to predict the presence of both G-quadruplexes and i-motifs from sequence (16). The scoring system of G4-iM Grinder is primarily designed G-quadruplexes, not specifically for i-motifs. In contrast, several machine learning-based tools have been developed for G-quadruplex prediction, including Quadron and G4Boost (17,18). Quadron employs a gradient boosting machine algorithm and sequence features to distinguish G4 motifs capable of forming stable structures, while G4Boost utilizes gradient-boosted decision trees to predict G4 folding probability and stability based on sequence and structural topology. However, given the distinct biophysical properties that differentiate G-quadruplexes from i-motifs, there is a critical need for the development of specialized i-motif searching software. Such software should integrate experimental data and specifically address the unique characteristics of i-motif forming sequences.

**Figure 1.**
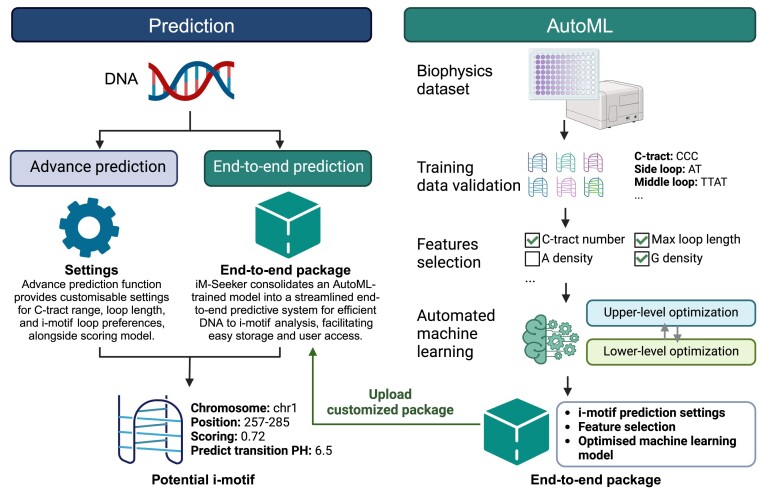
Schematic overview of iM-Seeker. The diagram illustrates the two primary functionalities of the server: ‘Prediction’, for direct i-motif detection and stability scoring, and ‘AutoML’, enabling users to train custom predictive models using their own i-motif biophysical data through automated machine learning. Created with Biorender.com.

To address this gap, we introduce ‘iM-Seeker’, an automated machine learning (AutoML) based platform for the prediction, scoring, and modelling of i-motifs (Figure 1). This system has been designed to predict potential i-motifs from DNA sequences or entire genomes by allowing the customisation of parameters. Moreover, leveraging AutoML methodologies, we provide users with the capacity to fine-tune the model by their own datasets and to procure end-to-end AutoML models tailored for i-motif scoring (Figure 1). In summary, our platform, ‘iM-Seeker’, aims to address the existing gaps in i-motif predictive software by introducing an advanced, customizable approach. We believe that these advancements can contribute positively to i-motif research, potentially facilitating new applications and insights in genomic science.

## Materials and methods

### Full-stack design of server

The ‘iM-Seeker’ webserver is designed to handle a wide range of tasks, which can be categorized into two main types: (i) computationally intensive tasks, such as automated machine learning (AutoML) modelling and genome-wide i-motif prediction and (ii) less computationally demanding tasks, including general requests and short DNA sequence predictions. Upon receiving a request, the server first assesses its computational complexity and assigns the task accordingly. For computationally intensive tasks, a sophisticated task queue model is employed at the backend. When a task is received, the backend generates a unique task identifier and returns it to the frontend. The task is then enqueued for processing, and its details, including execution status and results, are stored in a database. The frontend can use this unique task identifier to periodically check the backend for the status and final results of the task. On the other hand, for less computationally demanding tasks, the backend API immediately performs the necessary computations upon receipt and returns the results promptly. The frontend also includes a loading page to prevent accidental duplicate submissions. This intricate backend architecture is built using ‘Python3’, ‘fastAPI’ and ‘Celery’, with ‘Redis’ serving as the underlying database. Our servers are configured not to store any user data, ensuring complete data privacy. Additionally, all files generated by our predictive models are automatically deleted 30 days after creation. This information is displayed on our website as a reminder, and we encourage users to download their files within this timeframe to prevent data loss.

On the frontend, both the user interface and the underlying logic are developed using the ‘Vue3’ framework. The frontend dynamically routes different structures based on task identifiers to identify and instantiate the corresponding page templates, ensuring a seamless user experience. Furthermore, to maintain a smooth user experience and prevent any unintentional freezing, the frontend utilizes AJAX for asynchronous communication with the backend.

### Automated machine learning

**Figure 2.**
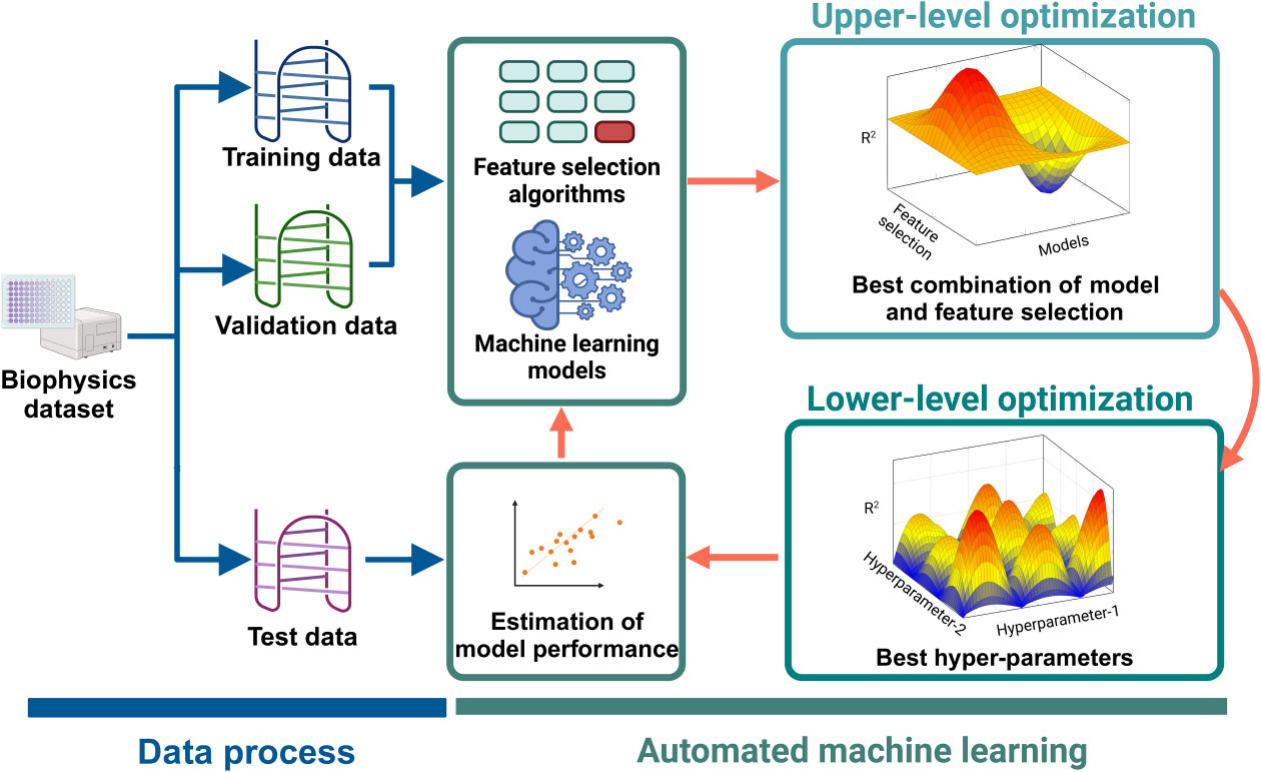
Automated machine learning modeling process. The schematic provides a visual overview of the ‘iM-Seeker’ framework, showcasing the integrated bi-level optimisation process for i-motif stability prediction. The lower-level involves hyper-parameter tuning via Tree-structured Parzen Estimator (TPE), while the upper-level employs Tabu search for model and feature selection. The process culminates in the evaluation of model performance based on testing *R*^2^ values, facilitating an automated approach for predictive model packaging and deployment. Created with Biorender.com.

The stability of i-motif structures is influenced by a complex interplay of various features. Thus, accurately predicting i-motif stability necessitates careful selection of relevant features, employment of an appropriate regression model, and optimal tuning of hyperparameters. To address this challenge, we developed ‘iM-Seeker AutoML’, an automated tool that systematically explores different combinations of feature selection methods, regression models, and hyper-parameter settings to discover the optimal configuration for i-motif stability prediction (Figure 2). By integrating these key components into an end-to-end pipeline, iM-Seeker enables de novo discovery of high-performing i-motif stability scoring models without the need for manual intervention or prior knowledge. This automated approach has the potential to significantly accelerate the development of accurate i-motif stability predictors and facilitate the investigation of these important non-canonical nucleic acid structures.

The overall architecture of our iM-Seeker AutoML system can be modelled as a bi-level programming problem (Figure 2). Bi-level programming is a mathematical framework in which one optimisation problem is nested within another in a hierarchical manner. In this framework, the outer optimisation task is referred to as the upper-level optimisation task, while the embedded optimisation task is known as the lower-level optimisation task. Mathematically, a bi-level programming problem can be formulated as follows:


(1) \begin{eqnarray*}\begin{array}{@{}*{2}{l}@{}} {\mathop {{\mathrm{maximize}}}\limits_{{{{{\bf x}}}^u} \in {{{\mathrm{\Lambda }}}^d} \times {{\mathbb{R}}^n},{{{{\bf x}}}^l} \in {{\mathbb{R}}^n}} }&{F\left( {{{{{\bf x}}}^u},{{{{\bf x}}}^{l{\mathrm{*}}}}} \right)}\\ {{\mathrm{\ subject\ to\ }}}&{{{{{\bf x}}}^{l{\mathrm{*}}}} \in {\mathrm{argmax}}\left\{ {{{f}_{{{{{\bf x}}}^u}}}\left( {{{{{\bf x}}}^l}} \right)} \right\}} \end{array}\end{eqnarray*}

where ${{{{\bf x}}}^u} \in {{{\mathrm{\Lambda }}}^d} \times {{\mathbb{R}}^n}$ and ${{{{\bf x}}}^l} \in {{\mathbb{R}}^n}$ denote the upper and lower-level variables, respectively. In particular, the upper-level variables consist of a combination of feature selection methods and regression models, which guide the overarching strategic decisions, including the selection of model structures and features. Meanwhile, the lower-level variables focus on the hyper-parameter settings for these models and methods, playing a crucial role in fine-tuning model performance (19). $F:{{{\mathrm{\Lambda }}}^d} \times {{\mathbb{R}}^n} \to \mathbb{R}$ and ${{f}_{{{{\mathrm{x}}}^u}}}:{{\mathbb{R}}^n} \to \mathbb{R}$ are the upper- and lower-level objective functions, respectively. Here we use the coefficient of determination, as objective function, denoted as ${{{{\bf R}}}^2}$ (20). Note that a bi-level programming involves nested optimisation/decision-making tasks at both levels. For any given ${{{{\bf x}}}^u}$, there is a corresponding pair $( {{{{{\bf x}}}^u},{{{{\bf x}}}^{l{\mathrm{*}}}}} )$ in which ${{{{\bf x}}}^{l{\mathrm{*}}}}$ is an optimal or near-optimal response to ${{{{\bf x}}}^u}$. This pair forms a viable solution to the upper-level optimization problem, assuming it adheres to all required constraints.

### Upper-level optimization

The AutoML part of iM-Seeker considers multiple feature selection methods as well as machine learning regression algorithms (Figure 2, Tables 1 and 2). In addition, we also provide the corresponding hyper-parameters associated with these feature selection methods and regression models, detailing their characteristics in the relevant tables. The goal of the upper-level optimization is to search for the best combination of all possible alternatives (84 in total) for the underlying regression task. For each candidate combination of feature selection and regression model, their corresponding hyper-parameter settings are optimised via a lower-level optimisation. At the upper level, the search of the best combination of feature selection and regression model is solved as a combinatorial optimisation problem as specified below.

**Table 1.** Overview of feature selection methods

Label	Algorithm	Parameter	Description
GUS	Generic Univariate Select	Mode, Score_func_name, Percentile, K_best	Selects features based on univariate statistical tests
SFM	Select From Model	Estimator_choice, N_estimators, Max_depth, Threshold, Max_features	Selects features based on importance weights from a model
SFS	Sequential Feature Select	Estimator, Scoring_choice, Forward_choice, K_features_value, N_estimators, Max_depth	Adds or removes features to form a feature subset
RFE	Recursive Feature Elimination	Estimator_name, N_features_to_select, Max_depth	Recursively removes features to minimize the feature set
RFECV	Recursive Feature Elimination with Cross-Validation	Min_features_to_select, Scoring, Estimator_name, Max_depth, N_estimators, Estimator	Performs RFE in a cross-validation loop to find the optimal number of features
VT	Variance Threshold	Threshold	Removes low-variance features

**Table 2.** Overview of machine learning models in iM-Seeker

Label	Algorithm	Parameter	Description
RIDGE	Ridge	Alpha	Linear regression with L2 regularization
DTR	Decision Tree Regressor	Max_depth, Min_samples_split, Min_samples_leaf	A non-parametric supervised learning method used for regression
RFR	Random Forest Regressor	N_estimators, Max_depth, Min_samples_split, Min_samples_leaf	A meta estimator that fits a number of classifying decision trees on various sub-samples of the dataset
GBR	Gradient Boosting Regressor	N_estimators, Learning_rate, Max_depth, Min_samples_split, Min_samples_leaf	A machine learning technique for regression problems, which produces a prediction model in the form of an ensemble of weak prediction models
SVR	Support Vector Regression	C, Kernel	Epsilon-Support Vector Regression
MLPR	Multi-layer Perceptron Regressor	Batch_size, Alpha, Learning_rate, Learning_rate_init, Momentum, Hidden_layer_sizes, Activation, Solver	A class of feedforward artificial neural network (ANN)
ETR	Extra Trees Regressor	N_estimators, Max_depth, Min_samples_split, Min_samples_leaf	Fits a number of randomized decision trees on various sub-samples of the dataset and uses averaging to improve the predictive accuracy and control over-fitting
BR	Bagging Regressor	N_estimators, Max_samples, Max_features, Base_estimator	A meta estimator that fits base regressors each on random subsets of the original dataset
ABR	Ada Boost Regressor	Base_estimator, Max_depth, N_estimators, Learning_rate	A meta estimator that begins by fitting a regressor on the original dataset and then fits additional copies of the regressor on the same dataset
STR	Stacking Regressor	Final_estimator_name, N_estimators, Max_depth, Max_iter, Learning_rate, Min_samples_leaf	Stacks the output of individual estimator and uses a regressor to compute the final prediction
HGBR	Hist Gradient Boosting Regressor	Max_iter, Learning_rate, Max_depth, Min_samples_leaf	A gradient boosting-based ensemble learning technique that operates on histograms to allow for faster learning
XGBR	XGBoost Regressor	Booster, Lambda, Alpha, Subsample, Colsample_bytree, Max_depth, Min_child_weight, Eta, Gamma, Grow_policy	Gradient boosted decision trees designed for speed and performance

#### Search space

For the upper level, the search space consists of all the valid combinations of feature selection and regression model picked up from the given portfolios (Tables 1 and 2). In practice, such portfolios can be amended and specified by the software engineers based on their preferences/requirements.

#### Objective function

Recall from the Equation (1), the objective function for the upper level ${{\bf F}}( {{{{{\bf x}}}^{\boldsymbol{u}}},{{{{\bf x}}}^{{\boldsymbol{l}}{\mathrm{*}}}}} )$ takes a combination from the portfolio (${{{{\bf x}}}^{\boldsymbol{u}}}$) and the optimized hyper-parameter of such combination $( {{{{{\bf x}}}^{{\boldsymbol{l}}{\mathrm{*}}}}} )$ as inputs. It then outputs the corresponding training ${{{{\bf R}}}^2}$ obtained by training model for comparison. Note that ${{{{\bf x}}}^{{\boldsymbol{l}}{\mathrm{*}}}}$ is initially unknown for a given ${{{{\bf x}}}^{\boldsymbol{u}}}$ at the upper-level before running a lower-level optimization routine. Therefore, the objective function at upper-level optimization is constrained and determined by the lower-level optimization.

#### Optimization algorithm

For the upper-level optimization, we use Tabu search to serve as the optimizer, which is also the entry point of the optimization phase (19).

### Lower-level optimization

The major purpose of the lower-level optimization is to identify the best hyper-parameters associated with the chosen combination of the feature selection method and the regression model (Figure 2, Tables 1 and 2). Specifically, this level is modelled and tackled as below.

#### Search space

At this level, the search space is the configuration space of the corresponding parameters for the feature selection and regression model picked up from the upper-level routine. Indeed, such a configuration space might be different depending on the chosen combination of feature selection and regression model (Tables 1 and 2).

#### Objective function

Recall from the Equation (1), when a combination of feature selection and regression model is picked up from the upper-level routine, the objective function for the lower-level ${\boldsymbol{f}}({{{{\bf x}}}^{\boldsymbol{l}}})$ takes the configuration of the corresponding hyper-parameters as the inputs $({{{{\bf x}}}^{\boldsymbol{l}}})$ and outputs the training ${{{{\bf R}}}^2}$ for the model. The ${{{{\bf R}}}^2}$ collected from the result of the low-level routine is finally used as the objective value at the upper-level routine to steer the optimization.

#### Optimization algorithm

It is not uncommon that the training and evaluation of a regression model is time consuming. To make our iM-Seeker computationally efficient, we apply the Tree-structured Parzen Estimator (TPE), a state-of-the-art Bayesian optimization algorithm for hyper-parameter optimzation of machine learning algorithms—as the optimizer for the lower-level optimization (21). Note that TPE can cope with a wide range of variables well, including integer, real, and categorical ones, which fits precisely with our requirements.

### iM-Seeker prediction function

**Figure 3.**
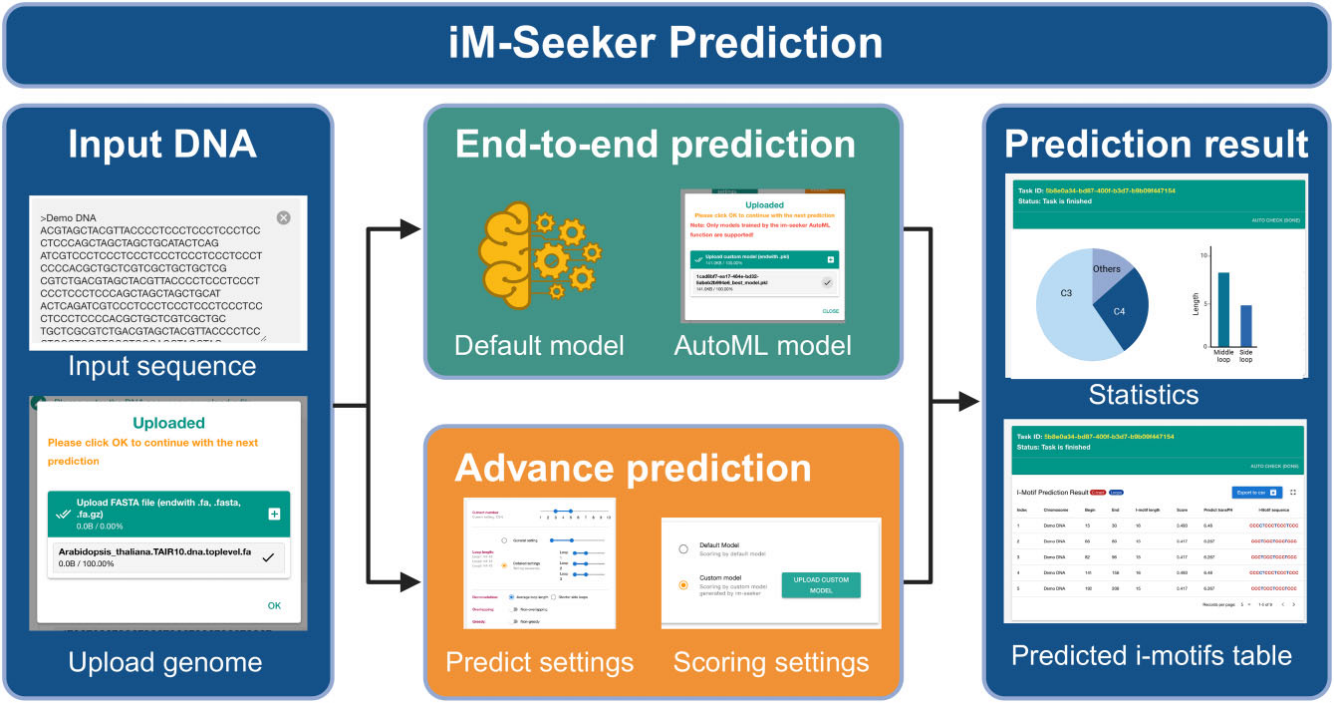
‘iM-Seeker Prediction’ function. A schematic representation of the i-motif prediction process, from DNA sequence input to the final prediction output. The process includes model selection, parameter customization, and a summary of predicted i-motifs with statistical analysis. Created with Biorender.com.

The ‘iM-Seeker prediction’ function offers several adjustable parameters (Figure 3), including the range of the number of cytosine tracts (C-tracts), loop length, the algorithm defining the loop, the allowance for i-motif overlap, and the option for a greedy algorithm. By default, the C-tract range is set from 3 to 5, allowing users to specify the number of C-tracts. The loop length can be adjusted in two ways: either by directly setting the overall loop range, e.g. 1–12, or by independently defining the lengths of the side and middle loops. The ‘Greedy algorithm’ determines the preference for longer (greedy) or shorter (non-greedy) i-motifs, with the default set to non-greedy. Each predicted i-motif is evaluated using a default machine learning model, providing results including an i-motif stability score and the predicted transition pH value (22). The aforementioned algorithm identifies the i-motif region, and to enhance feature extraction, we have incorporated an algorithm to precisely locate the loop (22). This feature provides two settings: striving for equal loop lengths or aiming for shorter side loops.

### iM-Seeker AutoML function

**Figure 4.**
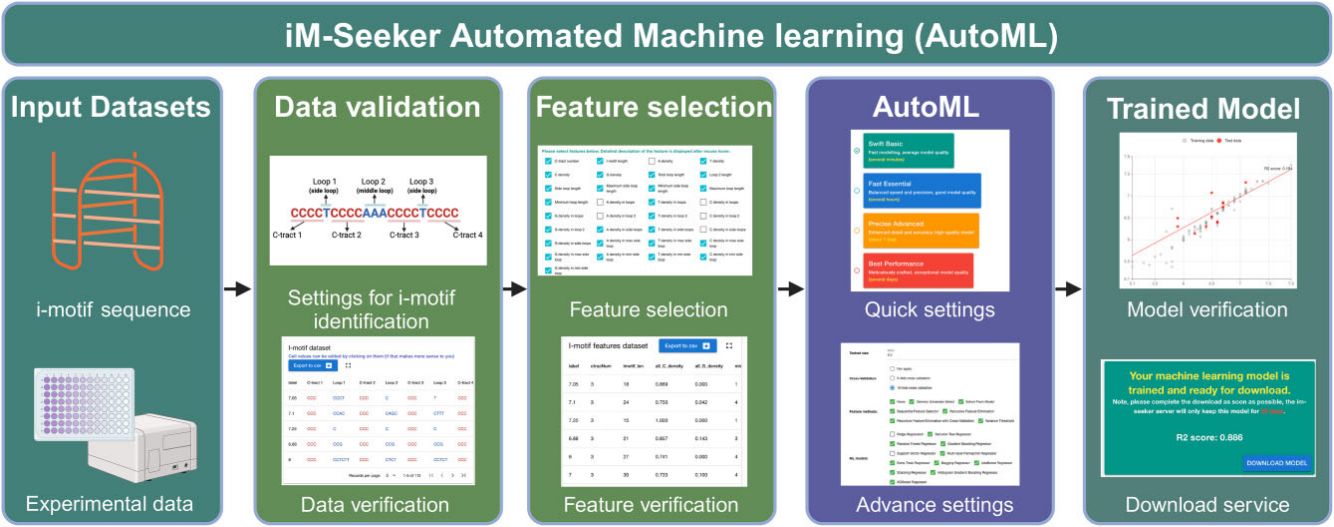
‘iM-Seeker AutoML’. This diagram illustrates the workflow from inputting i-motif sequences and experimental data to the final AutoML model verification. It details the data validation, feature selection and settings adjustment phases, concluding with a model performance graph and a prompt for downloading the trained machine learning model. Created with Biorender.com.

The ‘iM-Seeker AutoML’ function is a crucial function that streamlines the process of creating a comprehensive end-to-end predictive model for i-motif analysis (Figure 4). After training the model using automated machine learning (AutoML), iM-Seeker AutoML encapsulates it within a self-contained package, enabling users to easily progress from DNA sequence input to i-motif prediction and evaluation. The packaged dataset encompasses a variety of components: parameters for i-motif prediction including the C-tracts range and loop length; feature names chosen both by the user and AutoML; the corresponding extraction algorithms for these features; matrices for feature normalization alongside their associated parameters; and the machine learning model itself. These elements are subsequently transformed into byte streams, ensuring their standardized storage. Users can upload this package within the ‘iM-Seeker prediction’ function, thereby enabling a seamless DNA to i-motif prediction and evaluation experience.

## Main functionality of web server

The ‘iM-Seeker’ server, accessible at https://im-seeker.org, is an innovative, user-friendly computing platform dedicated to predicting DNA i-motif structures. It offers two main functionalities: ‘iM-Seeker Prediction’, enabling i-motif prediction and scoring from DNA sequences, and ‘ iM-Seeker AutoML’, facilitating the training of i-motif predictive models using custom data through automated machine learning (Figure 1). The download page provides resources such as i-motif predictions and scoring for common genomes (e.g. Human, Mouse, Arabidopsis), default models used by the server, and model training data. To ensure optimal usability and clarity for users, a comprehensive ‘Help’ section has been incorporated, providing detailed user guidelines. Each stage includes thorough guidance, instructions, and rigorous validation to prevent incorrect configurations.

### i-Motif prediction

The ‘iM-Seeker Prediction’ function identifies potential i-motifs in DNA sequences (Figure 3). The process unfolds in two stages: initially by specifying the DNA sequence and subsequently by adjusting prediction parameters. DNA fragments or multiple gene sequences can be pasted directly into the text input box or uploaded from local ‘Fasta’ files. After clicking the ‘continue’ button, the platform displays details of the detected sequence, such as sequence number, count, and length.

Within the ‘Settings’ for i-motif prediction, we provide two modes of prediction: ‘End-to-end prediction’ and ‘Advanced prediction’. There is no need for any complex parameterization in ‘End-to-end prediction’, we will use the common prediction parameters and default trained scoring model (22). Additionally, end-to-end prediction packages obtained in AutoML can be uploaded here to enable custom end-to-end prediction. ‘Advanced prediction’ allows users to customize processes, including prediction parameters (e.g. C-tracts, loop length) and selecting specific models.

Upon selecting the ‘Prediction’ function, the provided DNA sequence and specified settings are promptly processed on the server. As genome-level predictions can be time-consuming, these tasks are systematically queued. To facilitate monitoring of the task's progress, a unique task ID and related hyperlink are provided, revealing a progress bar when processing is still underway. This hyperlink can be securely stored, and results are readily accessible through its activation within a 30-day window following successful prediction. Upon task completion, a comprehensive table is displayed, showcasing the predicted i-motifs along with their pertinent attributes such as name, position, length, and sequence, with special attention given to C-tracts and loop regions. Additionally, each predicted i-motif is rigorously evaluated using a default machine learning model, providing insightful results including an i-motif prediction score and the predicted transition pH value. The results, displayed clearly and precisely, can be enlarged to full screen for detailed examination, and there is an option to download the associated data for further analysis or record-keeping.

### Automated machine learning modelling for i-motifs

A key function of the iM-Seeker server is automated machine learning (AutoML) (Figure 4). The current offerings include i-motif scoring models derived from UV molecular absorption-based i-motif transition pH data, labelled as the ‘Default’ model (22). Recognizing the potential limitations of this model in handling novel data or unique experimental conditions, ‘iM-Seeker AutoML’ allows for the integration of new data with existing datasets or the creation of entirely new models. This new dataset can be derived not only from the transition pH values but also from any quantifiable parameters associated with i-motif sequences, such as melting points under different pH conditions.

The ‘iM-Seeker AutoML’ function is meticulously designed to combine the creation of complex regression models, targeted feature extraction, and precise prediction algorithms into a comprehensive ‘end-to-end’ predictive system, tailored for specific dataset applications (Figure 4). The procedure begins with the input of DNA sequences representing i-motifs, along with their relevant characteristics such as transition pH and melting point, all formatted in CSV (comma-separated values). To ensure data integrity, the system thoroughly re-evaluates the input i-motif sequences, ensuring alignment with established i-motif prediction standards. Leveraging prior knowledge, 33 DNA i-motif-related features have been pre-determined. The framework provides the option to manually select essential features or to utilize the model's automated feature selection mechanism.

In the AutoML setup, the main focus is on defining the number of rounds and the scope of the model optimization search, including the number of computational rounds, iterations, model selection, and feature selection algorithms. These parameters are primarily used to regulate the balance between model runtime and performance. For user convenience, there are four preset parameter combinations to choose from, ranging from ‘Swift Basic’, which achieves average model quality in a few minutes, to ‘Best Performance’, which may take a few days but yields the optimal model. For those seeking more control, advanced settings allow for precise hyper-parameter fine-tuning, with detailed explanations provided in the Methods section. Initiating model training is straightforward, and given its resource-intensive nature, each training task is assigned a unique ID for progress tracking. Once completed, the model's performance metrics become accessible, alongside options to download the model, view statistical plots, and analyse training processes.

The AutoML configuration emphasizes carefully specifying the extent and depth of model optimization. This involves setting the number of optimization cycles, iterations, model selection, and feature selection algorithms. These parameters are crucial for achieving a balance between the model's execution time and its performance. The system offers four pre-configured parameter sets, ranging from ‘Swift Basic’ for rapid acquisition of average model quality in minutes, to ‘Best Performance’, a more time-intensive option that delivers the highest quality model over several days. For detailed control, advanced customisation options are available for precise hyper-parameter adjustments, with extensive documentation provided in the Methods section. The initiation of model training is designed to be efficient, and given its demanding nature, each training instance is assigned a unique identifier for tracking progress. Upon completion, the system provides access to a range of analytical tools, including performance metrics, options for model downloading, visual statistical representations, and in-depth insights into the training process.

### i-Motif densities in different species

**Figure 5.**
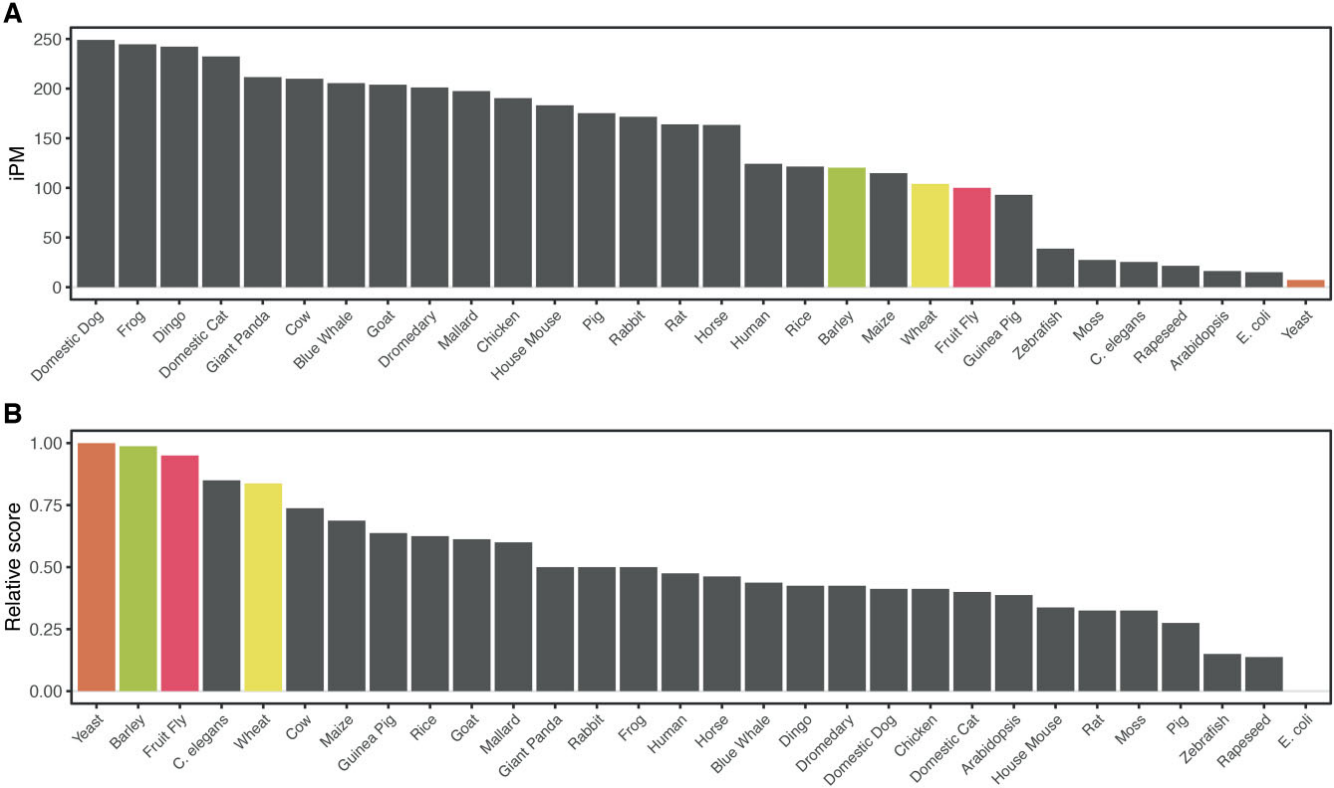
Statistics of DNA i-motifs in 30 species. (**A**) i-motif densities per million bases (iPM) across 30 species. (**B**) Relative average prediction scores of i-motifs in 30 species, obtained by normalizing the average scores of all predicted i-motifs in each species to a 0–1 scale. This normalization facilitates comparison across different datasets. Among the 30 species, four species with significant changes in rankings are highlighted.

Utilizing the comprehensive prediction capabilities of iM-Seeker server, we conducted a systematic analysis and scoring of DNA i-motifs across a diverse range of 30 species, encompassing mammals, plants, birds, fungi, and bacteria (Figure 5 and Table 3). Our methodology involved calculating the number of i-motifs per million nucleotides (iPM) for each species. This analysis revealed significant variability in i-motif densities. Notably, the genomes of the *Canis lupus familiaris* (domestic dog), *Xenopus tropicalis* (frog), and *Canis lupus dingo* (dingo) exhibited the highest i-motif densities, whereas *Escherichia coli* and *Saccharomyces cerevisiae* (yeast) displayed the lowest.

**Table 3.** i-Motif statistic of 30 species

Categories	General name	Latin name	Genome version	C density	iPM	Average score
Mammal	Blue whale	*Balaenoptera musculus*	mBalMus1.v2	0.202	205.523	0.401
	Cow	*Bos taurus*	ARS-UCD1.2	0.209	209.955	0.425
	Dingo	*Canis lupus dingo*	ASM325472v1	0.206	242.257	0.400
	Cat	*Felis catus*	Felis_catus_9.0	0.205	232.422	0.398
	Dog	*Canis lupus familiaris*	ROS_Cfam_1.0	0.205	249.056	0.399
	Dromedary	*Camelus dromedarius*	CamDro2	0.207	201.155	0.400
	Giant panda	*Ailuropoda melanoleuca*	ASM200744v2	0.205	211.594	0.406
	Goat	*Capra hircus*	ARS1	0.210	203.910	0.415
	Guinea pig	*Cavia porcellus*	Cavpor3.0	0.195	93.062	0.417
	Horse	*Equus caballus*	EquCab3.0	0.207	163.384	0.403
	House mouse	*Mus musculus*	GRCm39	0.203	183.174	0.393
	Human	*Homo sapiens*	GRCh38	0.194	124.276	0.404
	Pig	*Sus scrofa*	Sscrofa11.1	0.207	175.282	0.388
	Rabbit	*Oryctolagus cuniculus*	OryCun2.0	0.203	171.631	0.406
	Rat	*Rattus norvegicus*	mRatBN7.2	0.208	164.029	0.392
Plant	Arabidopsis	*Arabidopsis thaliana*	TAIR10	0.180	16.287	0.397
	Barley	*Hordeum vulgare*	MorexV3	0.222	120.359	0.445
	Maize	*Zea mays*	Zm-B73	0.234	114.865	0.421
	Moss	*Physcomitrium patens*	Phypa V3	0.166	27.428	0.392
	Rapeseed	*Brassica rapa*	Brapa 1.0	0.169	21.499	0.377
	Rice	*Oryza sativa*	IRGSP-1.0	0.218	121.502	0.416
	Wheat	*Triticum aestivum*	IWGSC	0.226	104.086	0.433
Bird	Chicken	*Gallus gallus*	GRCg7b	0.210	190.400	0.399
	Mallard	*Anas platyrhynchos*	ASM874695v1	0.209	197.513	0.414
Nematode	C. elegans	*Caenorhabditis elegans*	WBcel235	0.177	25.407	0.434
Bacteria	E. coli	*Escherichia coli*	ASM584v2	0.254	15.081	0.366
Amphibian	Frog	*Xenopus tropicalis*	UCB_Xtro_10.0	0.203	244.626	0.406
Insect	Fruit Fly	*Drosophila melanogaster*	BDGP6.32	0.208	100.107	0.442
Fungus	Yeast	*Saccharomyces cerevisiae*	R64-1-1	0.191	7.239	0.446
Fish	Zebrafish	*Danio rerio*	GRCz11	0.183	38.812	0.378

Furthermore, we evaluated these predicted i-motifs using the default scoring model of iM-Seeker, uncovering a notable divergence from the i-motif density rankings. Intriguingly, Hordeum vulgare (barley), yeast and Drosophila melanogaster (fruit fly), despite their lower i-motif density rankings at 19th, 22nd and 30th respectively, emerged with the top three average i-motif stability scores. This indicates a relative stability of i-motifs in these species. Such disparities in i-motif stability and density among species might be attributable to variations in their cellular environments and the distinct biological functions of the i-motifs within these contexts.

### Other functions

iM-Seeker performs i-motif prediction across standard genomes using various parameters. Relevant prediction data is accessible on the download page. By entering the species name or specific parameters in the search field, the system intuitively filters and presents pertinent data. Comprehensive guidelines for site navigation, along with in-depth explanations of i-motif prediction and AutoML parameters, are readily available on the help page.

## Discussion

The diversity of DNA structures beyond the conventional B-form has always been of interest, with particular emphasis on non-B structures like the i-motif. The discovery of the i-motif, which forms from cytosine-rich sequences under acidic conditions, revealed a unique quadruple-helical structure stabilized by hemiprotonated cytosine-cytosine base pairs. The potential implications of the i-motif in biological functions such as transcription modulation and telomere biology are profound, suggesting its value not only in understanding cell biology but also as a therapeutic target.

Addressing the previously identified gap in predictive software for i-motifs, ‘iM-Seeker’ emerges as a significant advancement, substantially contributing to the field of genomic science. This sophisticated platform uniquely combines precision, versatility, and user-centric design to facilitate accurate predictions, comprehensive scoring, and robust modelling of i-motifs. Underpinned by a meticulously curated dataset and cutting-edge machine learning methodologies, ‘iM-Seeker’ demonstrates an exceptional level of precision and adaptability.

The platform's intuitive interface ensures that users across various expertise levels can seamlessly navigate its extensive features and resources. The ‘iM-Seeker Prediction’ functionality empowers users to identify potential i-motifs within DNA sequences, offering a streamlined process that is both efficient and accurate. The ‘iM-Seeker AutoML’ functionality is a fully automated tool designed for training predictive models customized to the unique characteristics of each dataset. By bridging the gap in i-motif research and offering a platform, ‘iM-Seeker’ is poised to catalyse a new era of insights and advancements in genomic science.

## Data Availability

iM-Seeker is a freely accessible web server designed for the prediction and evaluation of i-motifs in DNA sequences. The iM-Seeker server is available at https://im-seeker.org.
